# Utilization of radiometric data for mapping primary and secondary sources of gamma radiation and radon/thoron release potentials in Ireland

**DOI:** 10.3389/fpubh.2024.1443332

**Published:** 2024-09-16

**Authors:** Mirsina M. Aghdam, Mark Kavanagh, Quentin Crowley

**Affiliations:** ^1^School of Natural Sciences, Trinity College Dublin, Dublin, Ireland; ^2^R&D Department, Geochron Ltd., Dublin, Ireland; ^3^Trinity Center for the Environment, Trinity College Dublin, Dublin, Ireland

**Keywords:** radon, thoron, drone surveys, radiation map, health

## Abstract

**Background:**

This paper presents a novel approach to predict and map radon and thoron levels. We developed separate radon and thoron prediction maps for Ireland and introduced a system for producing high-resolution 3D radiation maps which may be used for planning purposes in residential areas, recycling and demolishing waste depots, and quarries of building and construction material. Additionally, we highlight the critical need to monitor thoron alongside radon in indoor surveys, as thoron’s shorter half-life and higher energy levels may pose a greater health risk.

**Methods:**

We utilized Tellus radiometric survey data and indoor radon measurement records to investigate the spatial correlation between elevated indoor radon activity and anomalies in radioelement concentrations. We also estimated the degree of thoron interference in indoor radon surveys conducted in Ireland using CR-39 detectors. Field and laboratory surveys were performed to produce high-resolution radiation maps for four Irish quarries and estimate the radon and thoron potential of these quarries.

**Results:**

Our initial findings suggest that thoron may be the primary health issue in some parts of Ireland, surpassing radon. For example, our map shows that the expected thoron potential in county Donegal is significantly higher than that for radon. Our radon and thoron exhalation tests on building material samples from four random quarries confirm this. We also estimate that over 20% of the elevated indoor radon activity recorded by the EPA using CR-39 detectors may be attributed to thoron-related sources.

**Conclusion:**

This study contributes to a better understanding of the prevalence and impact of radon and thoron in Ireland, helping to determine the main radiological health issue related to indoor air quality in the country. Thoron’s impact on indoor air quality and health has been understudied in Ireland, necessitating more comprehensive studies and monitoring programs to accurately assess the prevalence and impact of both radon and thoron.

## Graphical abstract

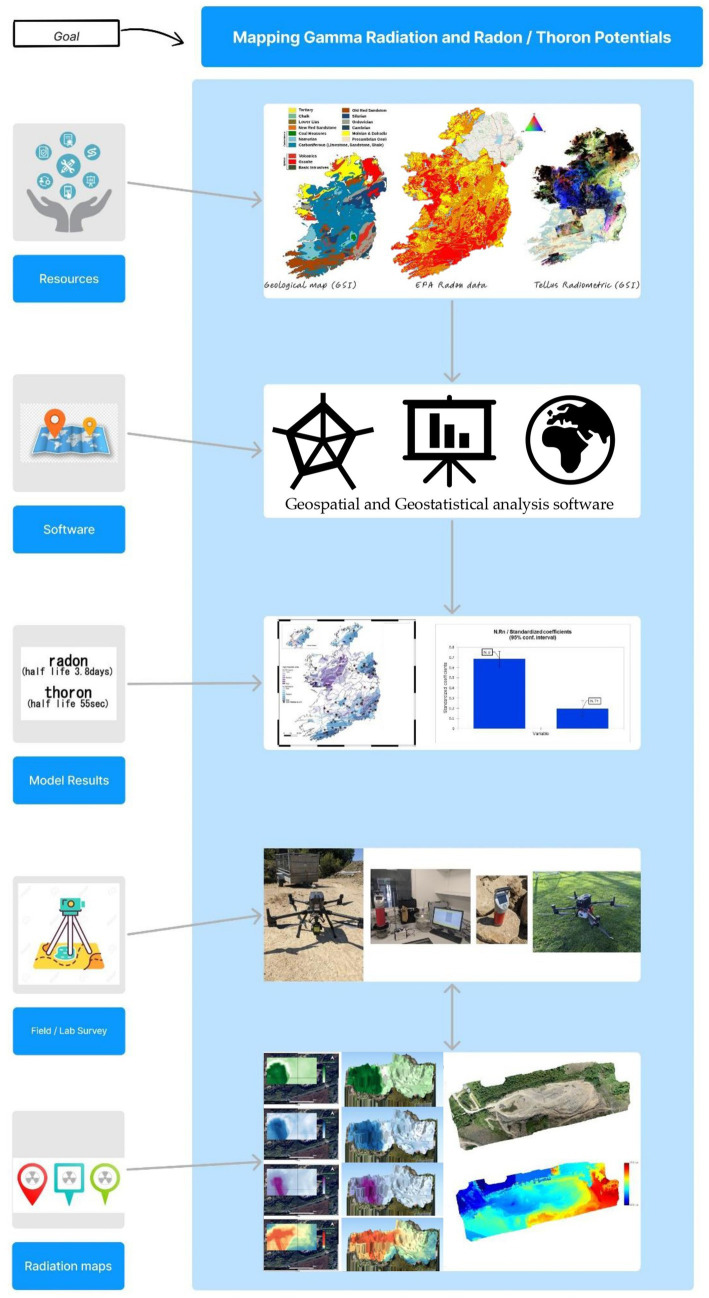

## 1 Introduction

Radon (^222^Rn) and thoron (^220^Rn) are naturally occurring radioactive gases that form from the decay of uranium and thorium in rocks and soil. Exposure to these gases can pose serious health risks such as lung cancer, with some evidence that they may also cause leukaemia and lymphoid cancers (1, 2). People with weak immune systems, especially children and older adults might be more vulnerable to the negative health effects of radon and thoron exposure. It is therefore vital to identify sources of radon and thoron in the environment. Unlike radon, thoron is not well studied or regulated both nationally and internationally. Exposure risk to thoron may be higher than radon in certain areas due to several factors; thoron has a shorter half-life compared to radon, which means it decays more quickly (3). This results in a higher rate of production and release of thoron gas into the environment. In fact, thoron is less dense than air, which affects its ability to disperse and travel long distances. Due to its lower density, thoron tends to rise and disperse more rapidly, which limits its horizontal spread in the atmosphere. Therefore most of the early research focused only on radon, as it was believed that radon was the primary source of indoor radiation exposure (4). This lack of knowledge and research on thoron contributed to its neglect and underestimation in dose calculations. The main source of risk associated with thoron exposure is its decay products rather than thoron itself (5, 6). The half-lives of thoron decay products are radium (^224^ Ra) (3.64 days), polonium (^218^ Po; 3.1 min), lead (^214^Pb; 26.8 min), and bismuth (^214^Bi; 19.9 min) (7). Thoron decay products have short half-lives but can still enter indoor spaces through cracks, like radon (8). Inside, they accumulate, attach to dust, and can be inhaled, posing health risks. Air circulation helps disperse thoron over longer distances (9).

Areas with high thoron potentials are more likely to be found where higher levels of thorium (^232^ Th) activity can be observed, such as zones with particular types of rocks (i.e., monazite, xenotime, and thorite-bearing rocks) or soils that contain thorium-bearing minerals. These rocks can sometimes have higher concentrations of thoron compared to radon. Additionally, certain building materials and construction practices can also contribute to higher thoron levels indoors (10). For example, some types of aggregates or natural stone (used as granite countertops and tiles, concrete blocks and slabs, red bricks, and quartzite/sandstone flooring) may contain higher amounts of thorium, leading to increased thoron emissions (11).

It is important to note that the specific levels of radon and thoron exposure can vary greatly depending on geogenic and geological factors and building characteristics (12). However, thoron is generally considered to have a higher risk of exposure than radon in many parts of the world. Therefore, it is recommended to conduct separate radon and thoron measurements, dose calculations and mapping in specific areas to correctly assess the levels of exposure (13).

Airborne radiometric data can be used to map the spatial distribution of radon and thoron potentials with high accuracy and resolution (14–16). By measuring the gamma radiation emitted by the decay products of radon and thoron, such as lead (^214^ Pb) and bismuth (^214^Bi), it would be possible to estimate the concentrations of radon and thoron in the air (17). These surveys help to identify areas with high radon and thoron potentials, which are essential to understanding the radiological health risks associated with indoor air quality.

In this study, we leverage Tellus radiometric data and geogenic information to create a preliminary model for predicting the risk of exposure to indoor radon. We then apply this model to estimate the degree of thoron interference in indoor radon measurements conducted by the Environmental Protection Agency of Ireland (EPA). The EPA’s surveys utilized CR-39 detectors to detect alpha particles, including those emitted by radon and its decay products. CR-39 detectors can be effective for thoron measurement, but their effectiveness depends on the specific type and setup. Some CR-39 detectors may not have the sensitivity needed to accurately differentiate between thoron and radon due to issues like nonuniform gas distribution and decay product homogeneity (18).

.To overcome these limitations, other detection methods and devices are often employed for thoron detection. However, it is worth noting that thoron interference can be a concern in areas with certain geological characteristics, such as high thorium content in rocks and soils. In regions where thoron interference is significant, it can affect the accuracy of radon measurements and risk assessments. Studies in part of Asia reported 20 to 40% thoron interference in indoor radon surveys (3, 19). To address this issue, it is necessary to use specific types of detectors [e.g., electret-based (20)], which can capture and measure the charged particles emitted by the thoron and its decay products more effectively. Researchers and regulatory bodies should consider the potential interference from thoron and consider appropriate corrective measures to ensure accurate radon measurements, precise dose calculations and reliable risk assessments (21).

As a result of the first part of this study, we prepared potential maps of the primary (soils and rocks) and secondary (building material) sources of radon and thoron based on the developed models. The second part of this study includes experiments to test the workability of the developed prediction maps and examine the feasibility of using a UAV system consisting of a drone, with a gamma detector, and lidar sensor to produce high-resolution radon, thoron, and gamma radiation potential maps at larger scales. For this purpose, we selected four natural stone quarries in County Donegal, Ireland for aerial surveys. We also used a handheld gamma-ray spectrometer with a 345 cm^3^ NaI detector in these field surveys to measure the activities of uranium, thorium, and potassium at specific points. Additionally, three representative rock samples were collected from each quarry and conducted follow-up laboratory measurements of radon and thoron exhalation rates using a radon and thoron monitor.

## 2 Materials and methods

### 2.1 Separate radon and thoron potential mapping and degree of thoron interference in indoor radon data

#### 2.1.1 Assumptions

Radon decays through a series of alpha and beta decays to eventually produce lead (^214^Pb). Similarly, thoron decays to produce bismuth (^214^Bi) through a series of decays. Assuming steady-state conditions, the activity of radon and thoron are correlated to the uranium and thorium concentrations estimated based on the decay products, lead (^214^Pb) and bismuth (^214^Bi) obtained using a radiometric survey (22–24). Thus, anomalous concentrations of uranium and thorium in the surrounding geology can lead to elevated indoor radon levels. It is important to note that while assuming a steady state for uranium and thorium is generally valid for airborne radiometric surveys, it does not mean that there are no variations or localized concentrations of uranium or thorium (25). However, the assumption simplifies the modelling and interpretation of the data collected during surveys, providing a useful framework for understanding the overall distribution of radioelements over large areas (26).

#### 2.1.2 Model setting

To build a model we introduced the number of anomalies in uranium (N. U) and thorium (N. Th) concentrations within each geological formation as the input variables and the number of anomalies in indoor radon values as the output parameter. The 100 K scale geological map of Ireland^1^ was used which identifies more than 1,100 individual formations. Uranium (U) is a ubiquitous element in the Earth’s crust, with an average concentration of approximately 2 ppm. In the context of assessing uranium concentrations, a value higher than 2 (ppm) (27) may be considered anomalous or elevated in certain circumstances. Th/U ratios are generally around 4 (28) so similar to uranium, the classification of a thorium concentration higher than 8 (ppm) may considered anomalous. Indoor values higher than the Irish national reference level (200 Bq.m^−3^) were defined as an anomaly. It is noteworthy that we also ran the models with hypothetical indoor radon anomaly thresholds of 100 and 300 Bq.m^−3^ but in both cases, no satisfactory results were obtained. Therefore, it seems that the Irish indoor radon threshold (i.e., 200 Bq.m^−3^) is suitable for indication of anomalies.

Ordinary least squares (OLS) regression analysis was successfully used in previous research to investigate the correlation between the number of anomalies in indoor radon and anomalies in uranium and thorium The description of the OLS regression model should cover key aspects such as:

- Model Assumptions: Linearity (linear relationships), Independence (independent observations), Homoscedasticity (constant error variance), Normality (normally distributed residuals), and No Multicollinearity (low correlation between predictors).
- Coefficients: Include the Intercept (value when predictors are zero), Slope Coefficients (change per unit change in predictors), Standard Errors (precision of estimates), and t-statistics and *p*-values (significance of coefficients).
- Interpretation of Results: Address R-squared (variance explained by the model), Adjusted R-squared (adjusted for the number of predictors), F-Statistic (overall model significance), Coefficient Interpretation (effect of predictors), and Residual Analysis (model assumption) (29).

XLS statistical add-in was employed to estimate regression coefficients (β₀, β₁, β₂) along with their standard errors, *p*-values, and measures of model fit (30). The goodness-of-fit of the regression model was examined using metrics like R-squared or adjusted R-squared. These measures indicate the proportion of variation in indoor radon that can be explained by the variables included in the model. Once the regression model was fitted, we calculated the estimated thoron interference for each data point by multiplying the thorium concentration by the corresponding coefficient (β₂) obtained from the regression analysis. This step provides an estimation of the impact of thoron on EPA’s indoor radon surveys.

#### 2.1.3 Mapping

The predicted number of anomalies in uranium and thorium values for each geoformation were obtained from fitting the regression model to ensure that these values represent the corrected or estimated indoor radon levels, accounting for the influence of uranium, thorium and geology in the model. We used statistical classification methods to categorize risk levels such as low risk, moderate risk, and high risk based on anomalies in uranium and thorium concentrations. According to this approach, to categorizing risk levels, first summary statistics for the number of uranium and thorium anomaly data, such as the mean, median, standard deviation, minimum, and maximum values were calculated to provide a general understanding of the distribution and range of the data and then based on that we defined three thresholds as follows: 1. *Low Risk*: N. uranium anomaly below 2 standard deviations from the mean and N. thorium anomaly below 1 standard deviation from the mean. 2. *Moderate Risk*: N. uranium anomaly between 2 standard deviations below and 2 standard deviations above the mean, or N. thorium anomaly between 1 standard deviation below and 1 standard deviation above the mean. 3. *High Risk*: N. uranium anomaly above 2 standard deviations above the mean or N. thorium anomaly above 1 standard deviation above the mean. QGIS software was utilized to plot the predicted corrected analogous values on a map, applying colour codes to represent different risk levels. This allowed us to visualize the spatial distribution of radon and thoron potentials of rocks and soils for the whole country.

#### 2.1.4 Secondary sources of radon and thoron

Also, the Tellus radiometric data was used to map the potential of building and construction materials with high radon and thoron. The map of quarries and mines in the shapefile format provided by Geological Survey Ireland (GSI) was used to identify the location of these features on a map of Ireland. A buffer zone with a 250 m radius surrounding every quarry was then applied. The average uranium and thorium (indicators of radon and thoron) were calculated for the point data located within the buffer zone. Finally, the locations with elevated values were displayed on the map to highlight the building and construction material sources that are most likely to contain high levels of radon and thoron.

### 2.2 Field and laboratory experiments

#### 2.2.1 Geological settings of the case study area

**Figure 1:**
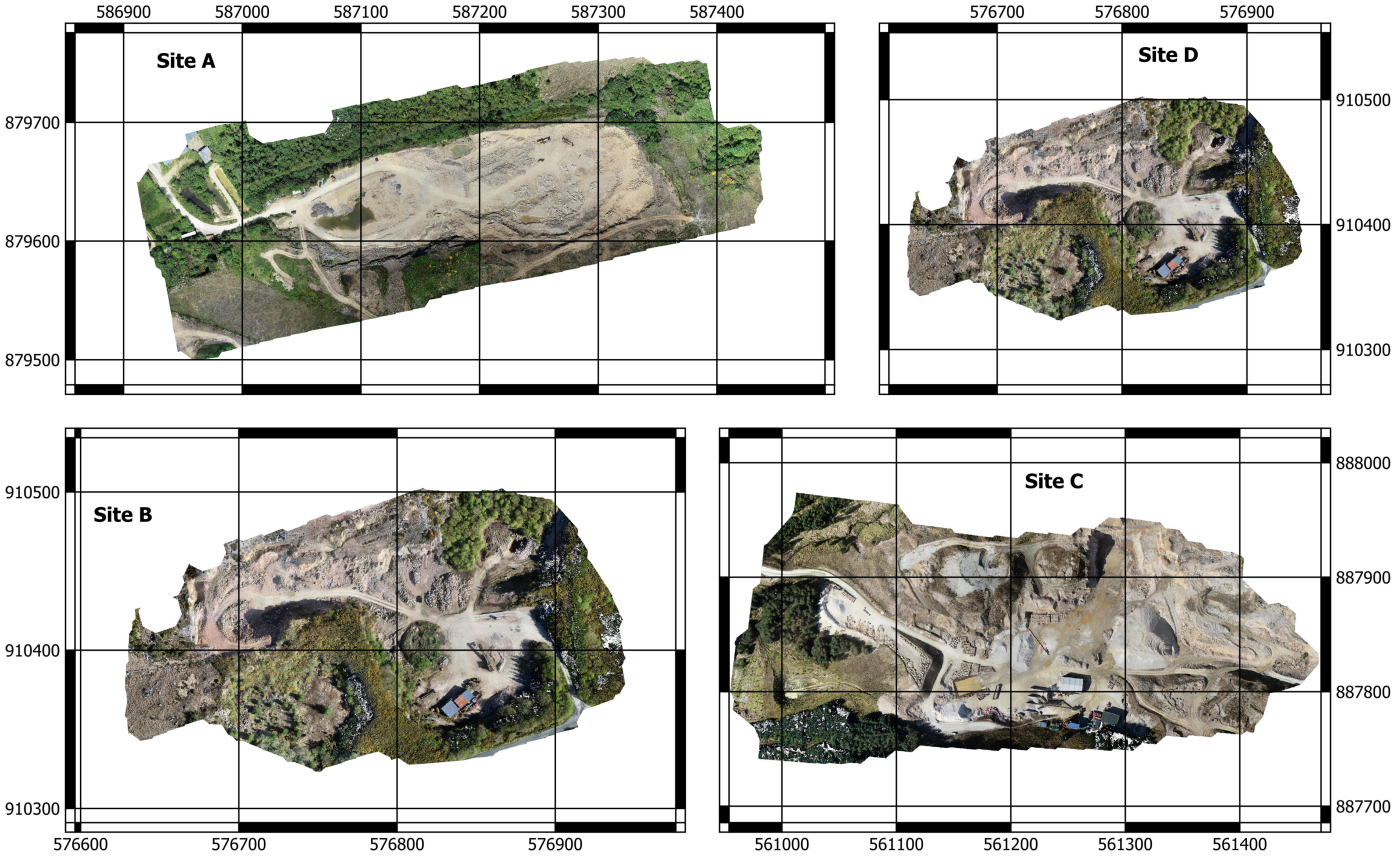
Map of the radiometric survey sites-County Donegal, Ireland.

The geology of the area is dominated by metamorphic rocks, particularly schists and gneisses, which have been formed through intense heat and pressure over millions of years and are made up of a mixture of sedimentary and volcanic rocks. The sedimentary rocks include sandstones, shales, and limestones, while the volcanic rocks include basalt and rhyolite. The Dalradian rocks have been subjected to intense heat and pressure over time, resulting in their metamorphism into schists and gneisses. These rocks are particularly well exposed in the Bluestack Mountains, which form a prominent range in south County Donegal. In addition to the metamorphic rocks, there were several granite intrusions in the area. These include the Diamond Granite, as well as other types such as Donegal Granite and Errigal Granite. Below is a brief description of the surveyed quarries (Figure 1) and their main products (31).

Site A\Mountcharles Sandstone Quarry: the main product of this quarry is sandstone composed of quartz, feldspar, and mica and has a grey colour due to iron oxide minerals. The rock probably formed around 400 million years ago during the Devonian period and is used as a building material for its durability and attractive colour.

Site B\Silver Quartzite Quarry: the Silver Quartzite quarry is located near Killybegs and is a popular source of this stone for construction projects in Ireland. The rock is silver-grey in colour with occasional streaks of pink and green. The original sedimentary rock was deposited during the Cambrian period. This rock type is also used as a building material for its durability and unique appearance.

Site C\Gold Quartzite Quarry: similar to silver quartzite, the product is often used for flooring, cladding, and paving due to its high resistance to wear and tear. It has a golden-yellow colour with occasional streaks of white and grey. The original sedimentary rock was deposited during the Cambrian period.

Site D\Diamond Granite quarry: located near Mountcharles and is often used for countertops, flooring, and paving due to its durability and resistance to heat and scratches. The unique pattern of Diamond Granite also makes it a popular choice for decorative purposes. It is part of the Donegal suite of granites, which were intruded in the Devonian period.

#### 2.2.2 Application of lidar sensor and radiation detector mounted on an unmanned aerial vehicle for detailed mapping in quarries

**Figure 2:**
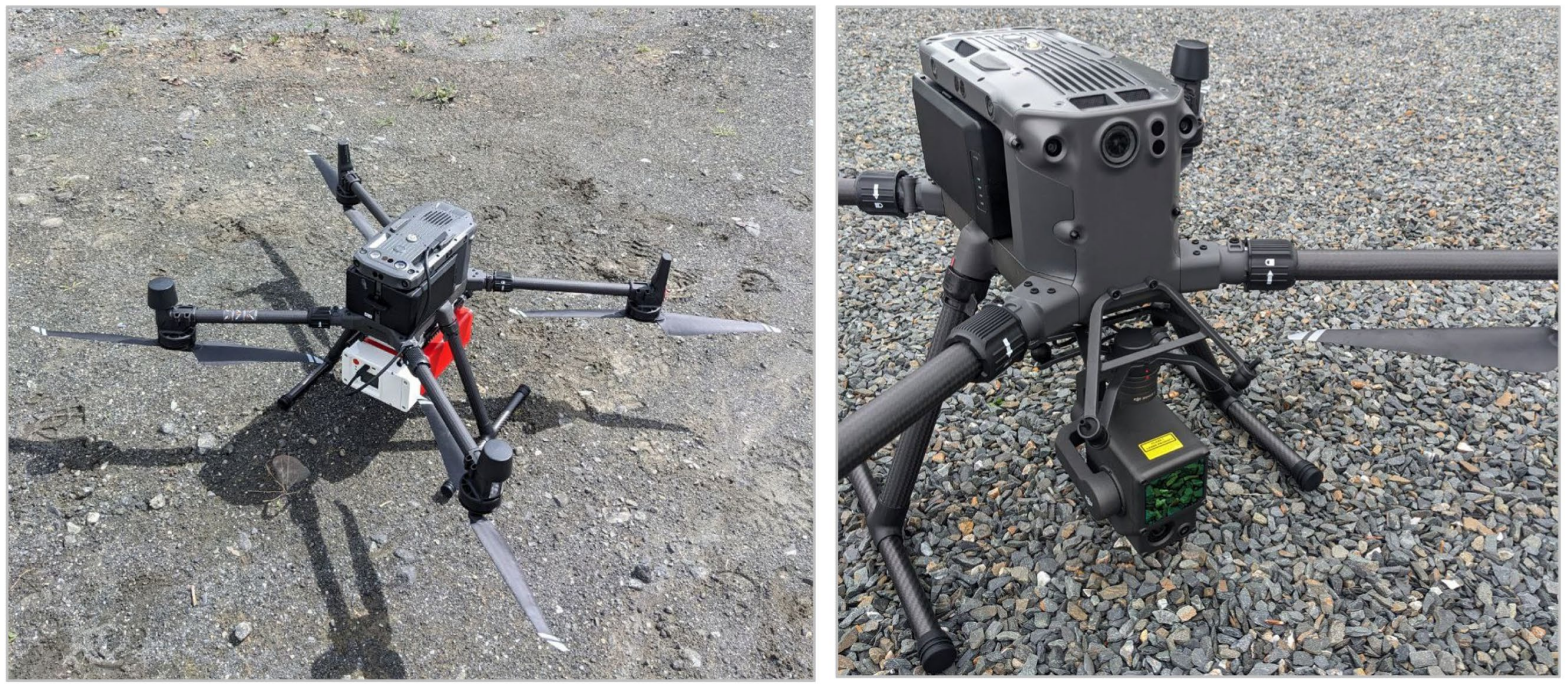
System for 3D radiation mapping including D230A gamma spectrometer (left) and Zenmuse L1 lidar sensor (right) mounted on a drone.

This innovative technology has revolutionized data collection in quarry environments, providing accurate and high-resolution information for radiation mapping purposes. The systems (see Figure 2) utilize remote sensing technology to capture precise elevation data, collect radiometric readings and generate highly detailed 3D models of quarry landscapes.

To create detailed 2D and 3D maps of the surveyed quarries using DJI Terra and data from Zenmuse L1 lidar, first, the data collected using the lidar system was transported to a laptop. The data is imported into DJI Terra, a software platform for mapping and surveying. In the workflow, lidar and photo data are processed separately. Photos are merged and orthorectified to create a 2D model. A 3D model is then produced using photogrammetry, which also generates a digital elevation model (DEM) for making a hillshade map. Lidar data is processed to produce a 3D point cloud model of the survey area.

The gamma radiation measurement device (D230A), which weighs about 3.5 kg, was attached to the bottom of the custom-made UAV quad-copter (Matrice 300 RTK) shown in Figure 2. The UAV-based radiometric measurements in quarries were made in two phases, with the first one in June 2023 and the second one in April 2024. The wind speed was low during the surveys (<1–2 m/s). A comprehensive survey route using 10 m line spacing resulting in a total line length of about 10 km was considered. The flight duration was 35–45 min for each quarry and each type of survey (i.e., radiation scanning and lidar mapping). For the lidar and photo data, the UAV altitude was 50 m and speed 10 m/s, except for the Diamond Granite quarry where proximity to a local airport necessitated a maximum 30 m flight altitude. The gamma surveys were conducted at between 25 and 30 m and between 2 and 3 m/s flight speed.

#### 2.2.3 Radon and thoron testing

**Figure 3:**
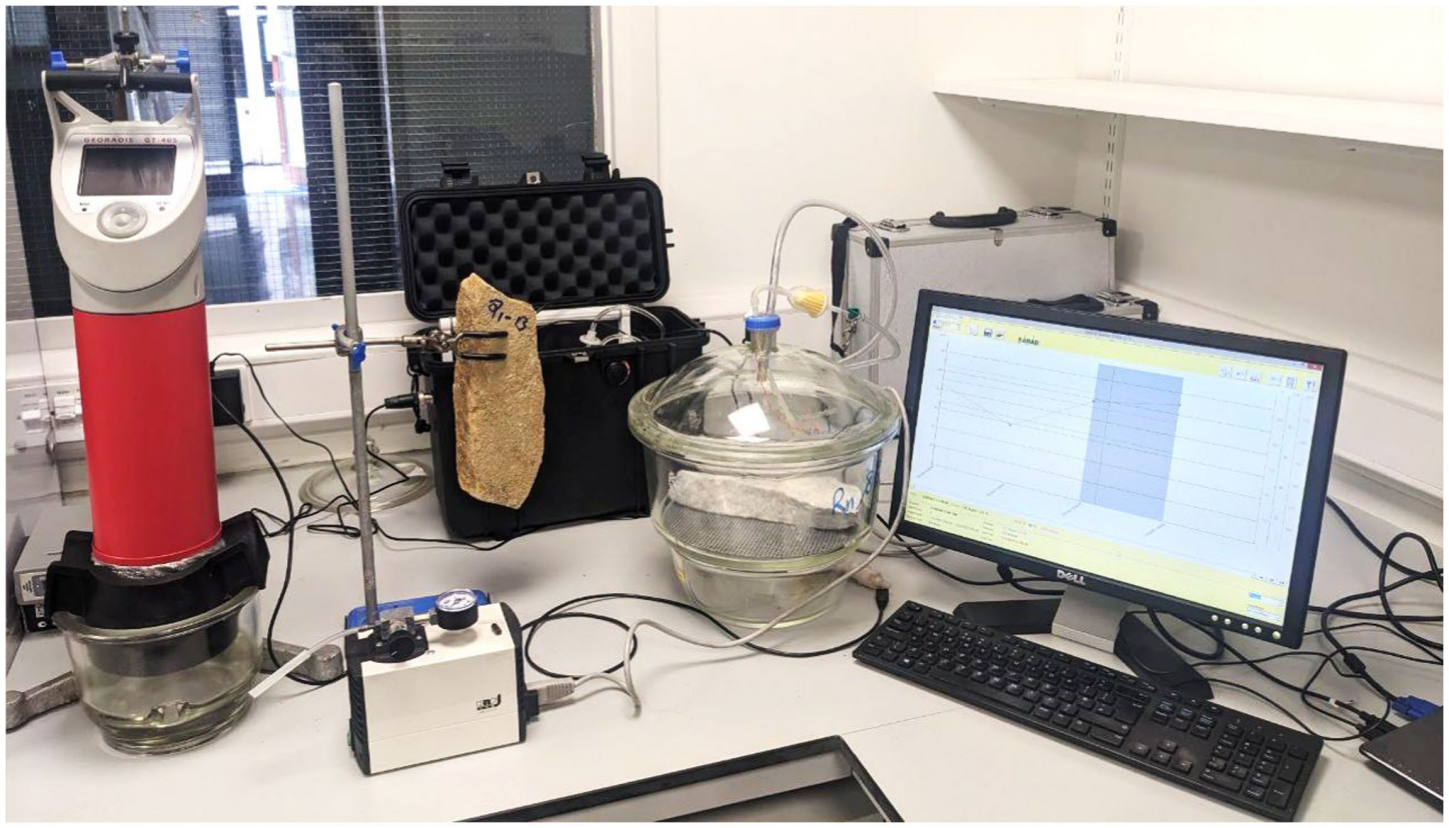
Radon/thoron and gamma measurement setup at Environmental Radioactivity Laboratory, Trinity Center for the Environment.

Three representative rock samples from each studied quarry were collected for radon and thoron testing using the RTM1688-2 Monitor Setup (Figure 3). The instrument comes with a DAkkS-compliant calibration certificate. The calibration process fulfils the requirements of the DIN ISO/IEC EN 17025:2018. The samples were placed in a vacuum desiccator container. It was ensured that the container was airtight to prevent any leakage of radon or thoron gases by using gas leak detector spraying. A small fan was put into the chamber to increase the air circulation rate. Sufficient time was allowed for radon and thoron equilibrium to be reached between the sample and the surrounding air (36–48 h of the testing period with a 1-h measurement interval).

**Figure 4:**
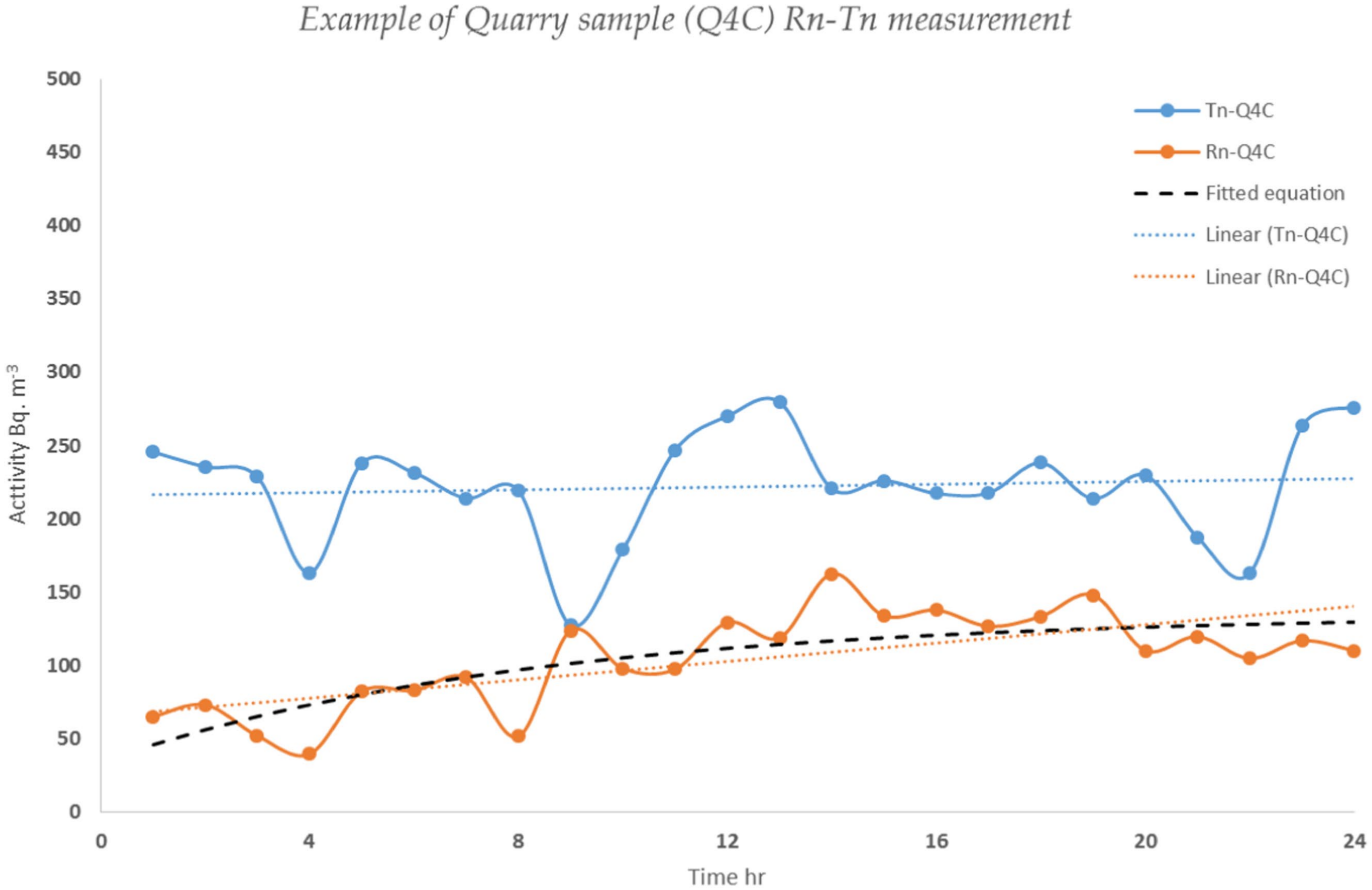
Radon and thoron growth models.

Considering Equations 1 and 2 (32) the exhalation rates of ^222^Rn (E^222^_Rn_, Bq m^−2^ h^−1^) and ^220^Rn (E^220^_Rn_, Bq m^−2^ h^−1^) were calculated by extrapolating the slope of the growth curve (m; Bq m^−3^ h^−1^) and the equilibrium ^220^Rn concentration (C_m_; Bq m^−3^) respectively. Figure 4 shows an example of ^222^Rn/^220^Rn concentrations as a function of time and the derived exhalation rates.


(1) $$E_{222Rn}=\frac{\left(m+\lambda_{222}\times C_{0}\right)\times V}{S}$$

(2) $$E220Rn=\frac{\lambda 220\times V0}{S}\frac{Cm}{e^{-\lambda 220\times \left(V1/Q\right)}}$$

Where λ_222_ and λ_220_ are ^222^Rn and ^220^Rn decay constants (h^−1^), C_0_ is the initial radon concentration (Bq m^−3^), V is the free total volume of the analytical system (m^3^), S is the surface of the sample, V_0_ and V_1_ (m^3^) are the free volume of the accumulation chamber and the volume between the outflow of the accumulation chamber and the inflow of the radon monitor, respectively. Q (15 L h^−1^) is the flow rate in the system.

#### 2.2.4 Gamma measurements

The GT-40 gamma-ray spectrometer in assay mode was used to measure the concentration of K (%), U and Th (ppm) in both collected rock samples and to collect the point-by-point data during the field survey. An Assay result for each test includes a full 1,024 channel spectra, survey data or scan data with GPS. The Geomon software package was then used for the graphical and numerical representation of the data and allowed data to be exported as a text file (CSV) for further processing.

## 3 Results

### 3.1 Radon and thoron mapping

#### 3.1.1 General statistics

The number of anomalies in indoor radon (>200 Bq.m^−3^), uranium (>2 ppm), and thorium (<8 ppm) observed within the area of each geological formation and their mean values were extracted using the “Zonal Statistics” in QGIS software. Table 1 shows the 10 formations with the highest average indoor radon anomalies calculated. While volcanic rocks are believed to show high radon potential, in the case of Ireland, the highest radon anomalies are observed in sedimentary and metamorphic rocks (i.e., shale, limestone and mudstone) rather than volcanic units. Shales, limestone and other types of sedimentary rocks often contain high levels of uranium-bearing minerals like Autunite (33). There can be a correlation between organic material and high uranium-bearing mineral contents in these formations, as organic-rich sedimentary rocks can contain higher concentrations of uranium. As can be seen from the table the indoor radon anomalies are associated with elevated uranium and thorium values (34). At the time of writing, the Tellus program does not cover the whole Irish territory yet. When the data becomes available it would be possible to have a better view of the radon and thoron-related anomalies in areas such as County Kerry and Limerick, in the south and southeast of the country.

**Table 1:** Indoor radon, uranium and thorium anomalies in geological units.

Geo code	Description	Unit name	U_Av. (ppm)	Th_Av. (ppm)	N.U	N. Th	N. Rn	Rn_Av. (Bq.m^−3^)
CDBURRFEI	Dolomitized limestone with shale	Fanore Member	NA	NA	0	0	5	1669.60
CDDRTG	Bioclastic cherty grey limestone	Dirtoge Limestone Formation	NA	NA	0	0	19	1196.68
CNWHIT	Sandstone & interbedded pyritic mudstone	White Strand Formation	2.29	8.42	217	1801	6	1081.33
CDBRICU	Bioclastic cherty limestone	Bricklieve Limestone Formation (upper)	2.37	NA	425	0	28	916.07
CDRKFD	Well-bedded argillaceous limestone	Rockfield Limestone Formation	NA	NA	0	0	44	915.73
CDCAHE	Crinoidal limestone & some nodular chert	Caherduggan Limestone Formation	NA	8.13	0	157	35	789.80
CDSNGLS	cyclical crinoidal limestone	Lissylisheen Member	NA	NA	0	0	16	751.63
CDSNGL	Cherty limestone	Slievenaglasha Formation	NA	NA	0	0	27	711.15
OCCAMPfv	Felsic volcanic	Campile Formation	2.12	9.20	235	11,121	119	693.45
CDRATH	Pale-grey massive mud-grade limestone	Rathronan Formation	2.49	8.29	781	17	17	692.71

#### 3.1.2 Regression model

**Figure 5:**
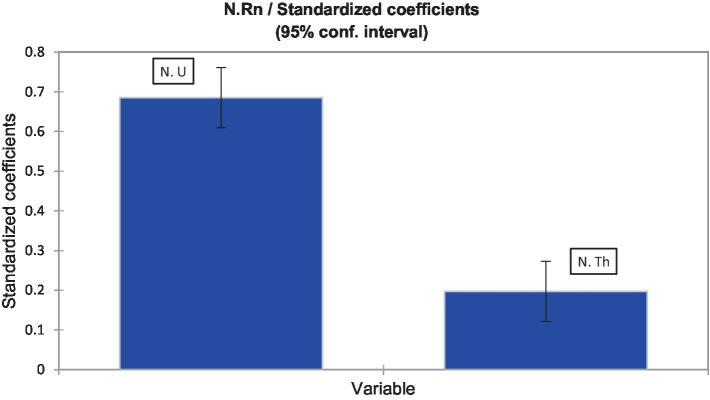
Contribution of uranium and thorium anomalies in elevated indoor radon activity (an indicator of the degree of thoron interference).

**Figure 6:**
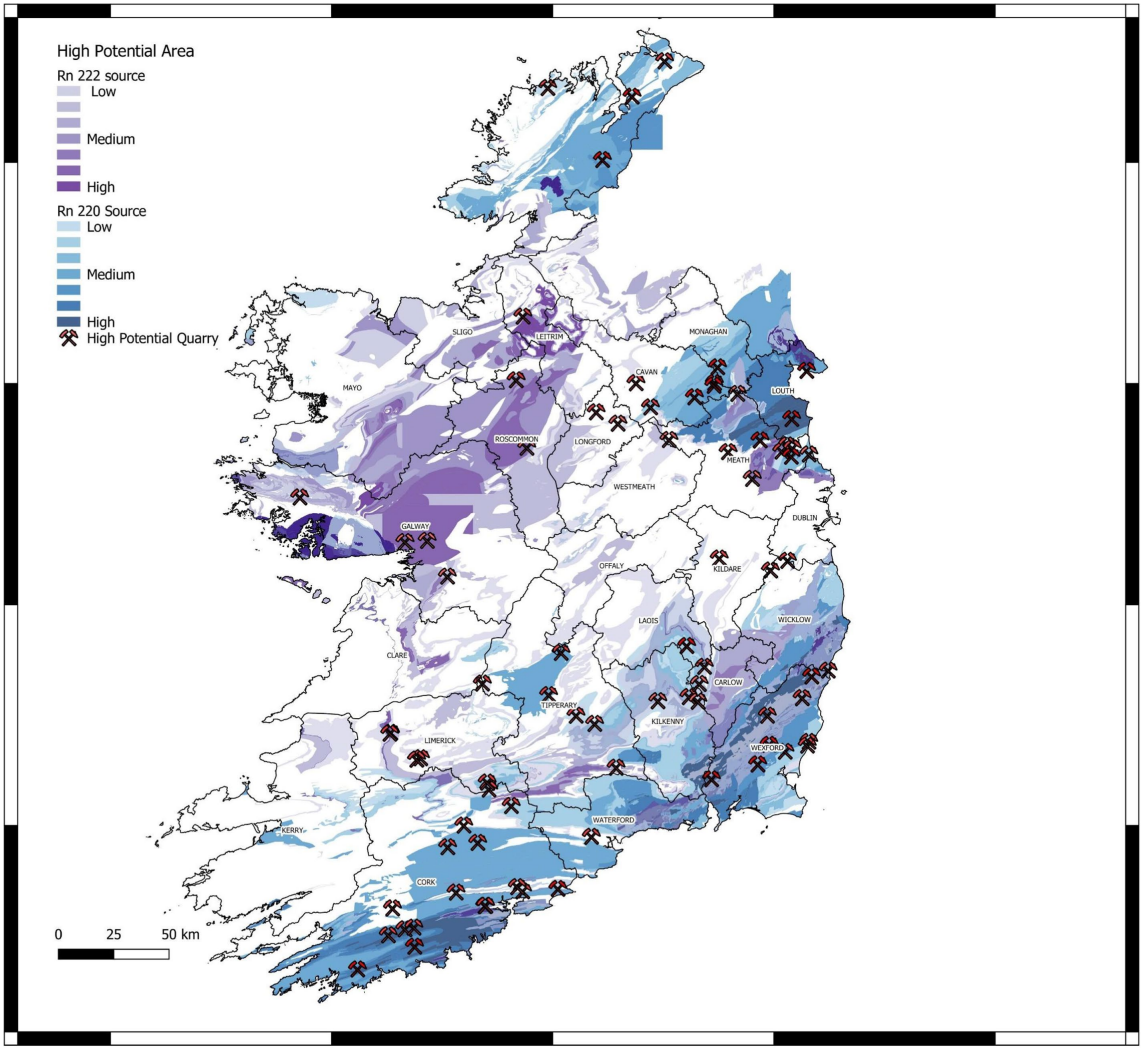
Geogenic radon and thoron potential map of Ireland.

In section 2.1, an Ordinary Least Squares (OLS) regression was used to develop models predicting the radon and thoron release potential of rock and soil formations (Figures 5, 6). Table 2 presents the goodness of fit coefficients of the model. The R^2^ (coefficient of determination) indicates that 51% of the variability in elevated indoor radon activity is explained by the uranium and thorium anomalies. The remaining variability is due to factors not included in this analysis. Key sources of uncertainty in the model include the lack of indoor thoron measurement data, limited indoor radon data coverage (only 10–15% of homes in Ireland are tested for indoor radon), and missing Tellus data for some parts of the country. To improve the model’s accuracy, better data coverage, indoor thoron tests, and inclusion of additional geogenic and anthropogenic parameters such as permeability and building properties would be necessary.

**Table 2:** Results of the regression model.

Model parameters (N.Rn)							Goodness of fit statistics	
Source	Value	Standard error	t	Pr > |t|	Lower bound (95%)	Upper bound (95%)	DF	326.00
Intercept	9.07	1.21	7.52	**< 0.0001**	6.70	11.45	R^2^	0.52
N.U	0.06	0.003	17.77	**< 0.0001**	0.055	0.069	Adjusted R^2^	0.51
N. Th	0.003	0.001	5.11	**< 0.0001**	0.002	0.004	MSE	455.06
Standardized coefficients (N.Rn)							RMSE	21.33
Source	Value	Standard error	t	Pr > |t|	Lower bound (95%)	Upper bound (95%)	MAPE	466.11
N.U	0.686	0.039	17.768	**< 0.0001**	0.610	0.761	DW	1.35
N. Th	0.197	0.039	5.111	**< 0.0001**	0.121	0.273	Cp	3.00
Correlation matrix				Summary statistics			AIC	2016.61
Variable	N.U	N. Th	N.Rn	Variable	Range	Mean ± Std. deviation	SBC	2028
N.U	**1**	0.023	0.690	N.Rn	1–356	12.72 ± 30.59	PC	0.49
N. Th	0.023	**1**	0.213	N.U	0–9,549	39.81 ± 338.28	Press	203222.87
	0.690	0.213	**1**	N. Th	1–33,869	458.45 ± 2326.63	Q^2^	0.34

*N.Rn: number of indoor radon anomalies, N. U: number of uranium anomalies, and N.Th: number of Thorium anomalies. Bold values denote statistical significance at the *p* < 0.05 level.

Given the extremely low probability corresponding to the t value (Table 2), it can be confidently assumed that the null hypothesis (no effect of uranium and thorium anomalies) is incorrect. Therefore, it is concluded that the uranium and thorium values significantly contribute to the indoor radon prediction model. As shown in Figure 5, thorium anomalies (thoron indicator) can account for around 20% of elevated indoor radon levels due to radon-thoron discrimination issues by CR-39 detectors used in indoor radon surveys in Ireland. This degree of thoron interference in indoor radon measurements is consistent with previous findings in Southeast Asia. The standardized coefficients and model parameters in Table 2 can be used to reproduce similar outputs. According to the methodology introduced in section 2.1.3, we produced a map of radon and thoron potential for Ireland’s geological formations (Figure 6). This is the first attempt to distinguish between geogenic radon and thoron potentials, re-evaluate radon and thoron dose exposure, and better estimate the health risks associated with potential residential radon and thoron exposure.

### 3.2 Field surveys and laboratory test campaign

#### 3.2.1 Radon/thoron exhalation rates and radionuclide concentrations

Table 3 represents the results of radon and thoron testing of the collected samples. For site A, the sandstone quarry, very low radon and low thoron exhalation rates (mean values of 0.045 and 560.53 Bq m^−2^ h^−1^, respectively) were measured. Sandstone has normally low radon and thoron levels because it is a sedimentary rock that is typically formed from sand-sized mineral particles, such as quartz, feldspar, and mica. These minerals do not contain significant amounts of uranium or thorium. The low mean U and Th values (0.53 and 3.62 ppm) measured at the site also justify the low radon and thoron activity of sandstone samples. The Th/U ratio can be used to study alteration in rocks. During alteration, the Th/U ratio can change due to the mobility of thorium and uranium isotopes. For example, if a rock is altered by hydrothermal fluids that contain high concentrations of uranium, the Th/U ratio in the rock may decrease. Conversely, if a rock is altered by fluids that contain high concentrations of thorium, the Th/U ratio may increase. Also, a high Th/U ratio can indicate that the rock is relatively young, while a low Th/U ratio suggests that the rock is much older (35). A Th/U ratio of 6.83 suggests that the rocks of this site are rather young and have probably undergone an alteration process. Also, a rather high amount of K in sandstone (mean value of 4%) can be because of the specific geological conditions that caused the enrichment of organic matter and potassium-40. Sources of gamma radiation in sandstone can often be attributed to clay minerals, potassium feldspars (in arkosic sandstones), micas (in micaceous sandstones) or heavy minerals (such as zircons) (36).

**Table 3:** Field and laboratory test results.

Test type	Code	Note/Location	Origin	Type	Exhalation rates of 220Rn (E220Rn, Bq m^−2^ h^−1^) ± error		Exhalation rates of 222Rn (E222Rn, Bq m^−2^ h^−1^) ± error		**U** ppm	Th ppm	K %	Dose rate nSv h^−1^
Lab samples	Q1A	White	Site A	Sandstone	182	59	0.07	0.03	1.65	2.45	0.76	26.53
	Q1B	Brown			943	75	0.04	0.01	1.93	2.58	0.79	27.54
	Q1C	Gray			557	70	0.03	0.02	1.41	2.28	0.76	25.05
Field assay	I-1	54.664896–8.197531833			NA				0.48	3.09	4.10	62.14
	I-2	54.664896333–8.197524833							0.41	3.63	3.66	57.10
	I-3	54.664844167–8.197926833							0.70	4.14	4.24	66.61
Lab samples	Q2A	Silver	Site B	Quartzite	1,238	86	0.03	0.02	1.68	2.55	0.94	28.49
	Q2B	Amphibolite			103	24	0.06	0.02	1.60	2.57	0.83	27.26
	Q2C	Micaceous Pegmatite			1,547	107	0.23	0.03	2.11	2.14	0.72	26.76
Field assay	II-1	54.746027833–8.559790167			NA				0.93	4.18	2.74	50.02
	II-2	54.746156333–8.560222667							0.92	4.42	3.05	54.60
	II-3	54.7455685–8.560646333							1.30	5.64	2.94	57.30
Lab samples	Q3A	Gold	Site C	Quartzite	6,091	145	0.07	0.01	1.80	2.78	0.72	27.03
	Q3B	Dark Gray			1743	127	0.05	0.02	2.03	2.27	0.65	26.41
	Q3C	Blackstone			599	110	0.07	0.05	1.98	2.26	0.74	27.40
Field assay	III-1	54.737670833–8.600628			NA				1.03	3.08	2.54	46.27
	III-2	54.737426–8.600483833							0.71	3.87	2.46	44.79
	III-3	54.737381833–8.601956667							0.68	2.78	1.22	28.34
Lab samples	Q4A	Weathered	Site D	Granite	3,717	148	0.20	0.02	1.76	2.68	0.73	27.21
	Q4B	Weathered			3,669	214	0.38	0.08	1.75	3.19	0.84	29.35
	Q4C	Fresh rock			1945	143	0.58	0.11	1.71	2.71	0.74	27.37
Field assay	IV-1	54.940864833–8.363611333			NA				15.09	9.31	14.57	265.44
	IV-2	54.941109833–8.362401833							6.37	6.18	4.08	98.04
	IV-3	54.941283667–8.360480333							15.53	14.38	6.68	189.86

For site B, the silver quartzite quarry, the radon exhalation rates are very low (mean value of 0.11 Bq m^−2^ h^−1^) but we see elevated thoron activities (1238.45 and 1547.07 Bq m^−2^ h^−1^) except for one of the samples which was made of amphibolite with very low thoron activity. Amphibolites are typically formed through the metamorphism of basaltic rocks, which generally do not contain high levels of thorium and thoron (36). The average U, Th and K values measured at the site were 1.05 ppm, 4.75 ppm and 2.91%. A Th/U ratio slightly higher than 4 suggests that a low degree alteration may have occurred.

For Site C, the silver quartzite quarry, a similar range of radionuclide and radon/thoron activity was observed except for one of the samples for which the highest thoron exhalation rate of 6090.64 Bq m^−2^ h^−1^ was recorded the mean radon and thoron exhalation rates were 0.03 and 2810.79 Bq m^−2^ h^−1^, respectively. The average U, Th and K values measured at the site were 0.81 ppm, 3.24 ppm and 2.07%. Similarly, a Th/U ratio slightly higher than 4 suggests that a low degree alteration may have occurred.

On the southern and eastern sides of site D, the granite quarry was highly weathered resulting in high uranium content around 15 ppm and 10% potassium activity. As thorium isotopes have lesser mobility than uranium and potassium, the measured thorium activity was not high (around 12 ppm at altered zones). The radon and thoron exhalation rates of samples collected from altered zones are two times those sampled from fresh rock. This is because of enrichment/depletion processes as a result of the complex metamorphic history, alteration and/or weathering (37). The E_220Rn_ from the fresh sample was medium to high level (1944.68 Bq m^−2^ h^−1^), but E_222 Rn_ is still low to medium.

A rough comparison between the measured radon and thoron exhalation rates and the geogenic radon-thoron levels predicted in Figure 6 (i.e., very low radon and thoron potential for site A, low radon and low to medium thoron potentials for site B and C and medium thoron and radon potential for site D) shows that the predicted values agree with those measured even at a small scale.

The exhalation rates measured here suggest that thoron may pose a greater risk than radon from the samples collected from Donegal quarries. According to recent research (6, 11, 13), nowadays it is an accepted fact that thoron activity needs special attention in high background radiation areas. Although we expect the total effective dose from these materials would most likely be lower than the threshold level of 1 mSv per year, the shorter half-life and higher ionizing potential of thoron may make it a greater concern from the health point of view. It is noteworthy that the health risks associated with thoron arise primarily from its decay products, which have a higher ionizing potential and can pose significant concerns in certain environments.

#### 3.2.2 Lidar mapping

**Figure 7:**
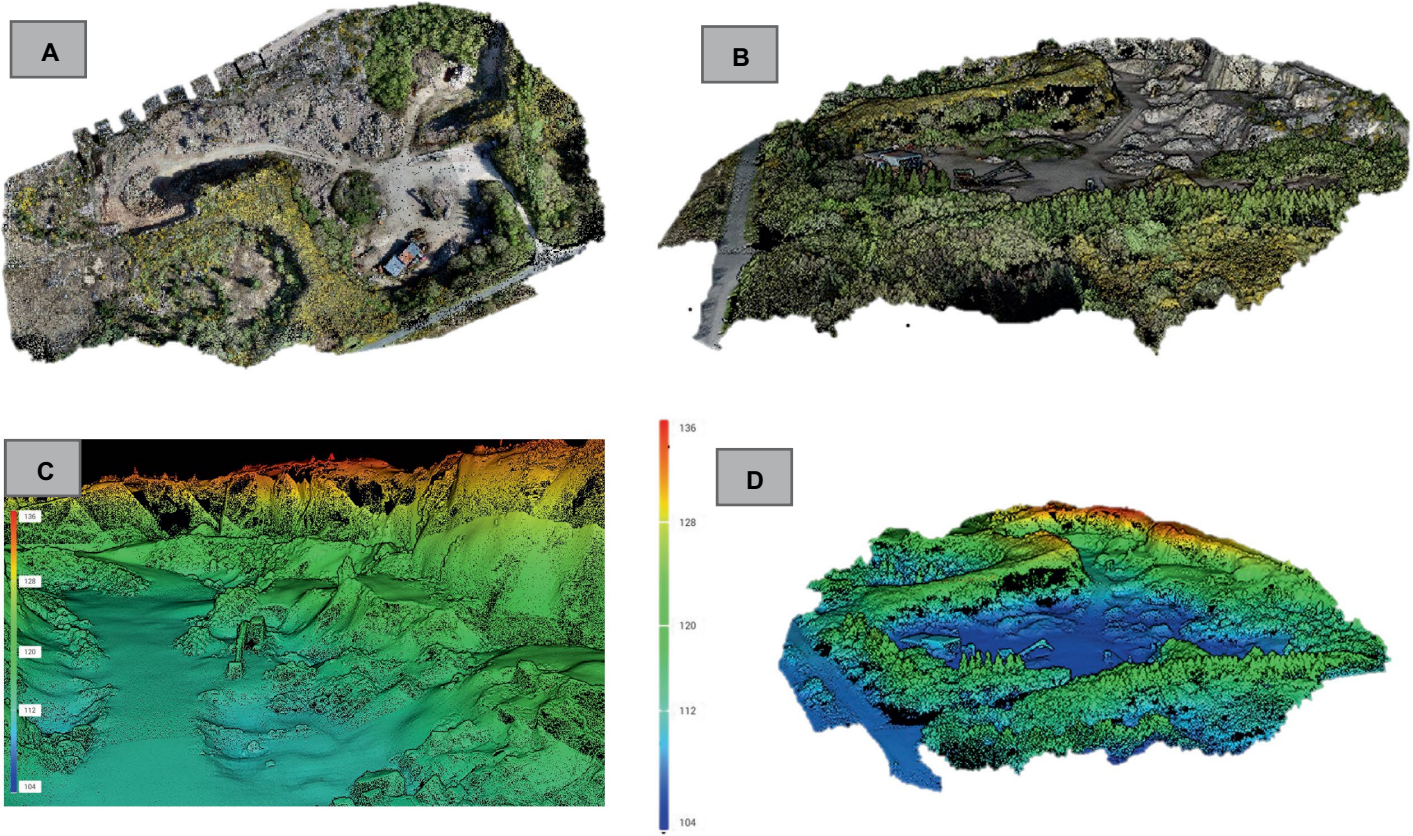
**(A)** 2D and **(B)** 3D photogrammetric scans. **(C)** Lidar point cloud in map view. **(D)** Lidar point cloud in oblique view of the Diamond Granite Quarry showing height above sea level in meters.

To generate a Digital Elevation Model (DEM) and extract elevation information for the simulations and radiation mapping, a drone-borne lidar was used. The Zenmuse L1 integrates a Livox lidar module, a high-precision IMU, and a 1-inch CMOS camera on a 3-axis stabilized gimbal. When used with the Matrice 300 RTK and DJI Terra, it offers a complete solution for real-time 3D data capture. This setup efficiently captures detailed structural features and produces highly accurate reconstructed models. 2-D and 3-D point cloud data position (Figure 7) and orientation estimates provided by the lidar sensors were used for simultaneous localization and mapping which enabled to estimation of positions of radiation hot spots with centimetre accuracy and orientation in real-time (38).

#### 3.2.3 Radiation maps

Empirical Bayesian kriging (EBK) is a geostatistical method that was used to produce distribution maps of various parameters, including uranium, thorium, potassium, dose rate, total gamma, and error of prediction maps (39). The process involved data preparation by importing the radiometric and DEM data obtained from drone surveys into ArcGIS and ensuring that they were in the correct format and coordinate system. Exploratory data analysis was then performed to understand the spatial distribution of the data and identify any outliers or anomalies using various tools in ArcGIS such as histograms, scatterplots, and spatial autocorrelation.

After analyzing the data, an EBK model was fitted to it by selecting a variogram model that described the spatial correlation between the data points and estimating the model parameters using maximum likelihood estimation. The accuracy of the model was then assessed using cross-validation by splitting the data into training and validation sets and using the training set to fit the model and the validation set to test its accuracy. Finally, distribution maps of the various parameters of interest were produced using EBK by specifying the input data, the EBK model, and any additional parameters such as the search radius and output cell size.

**Figure 8:**
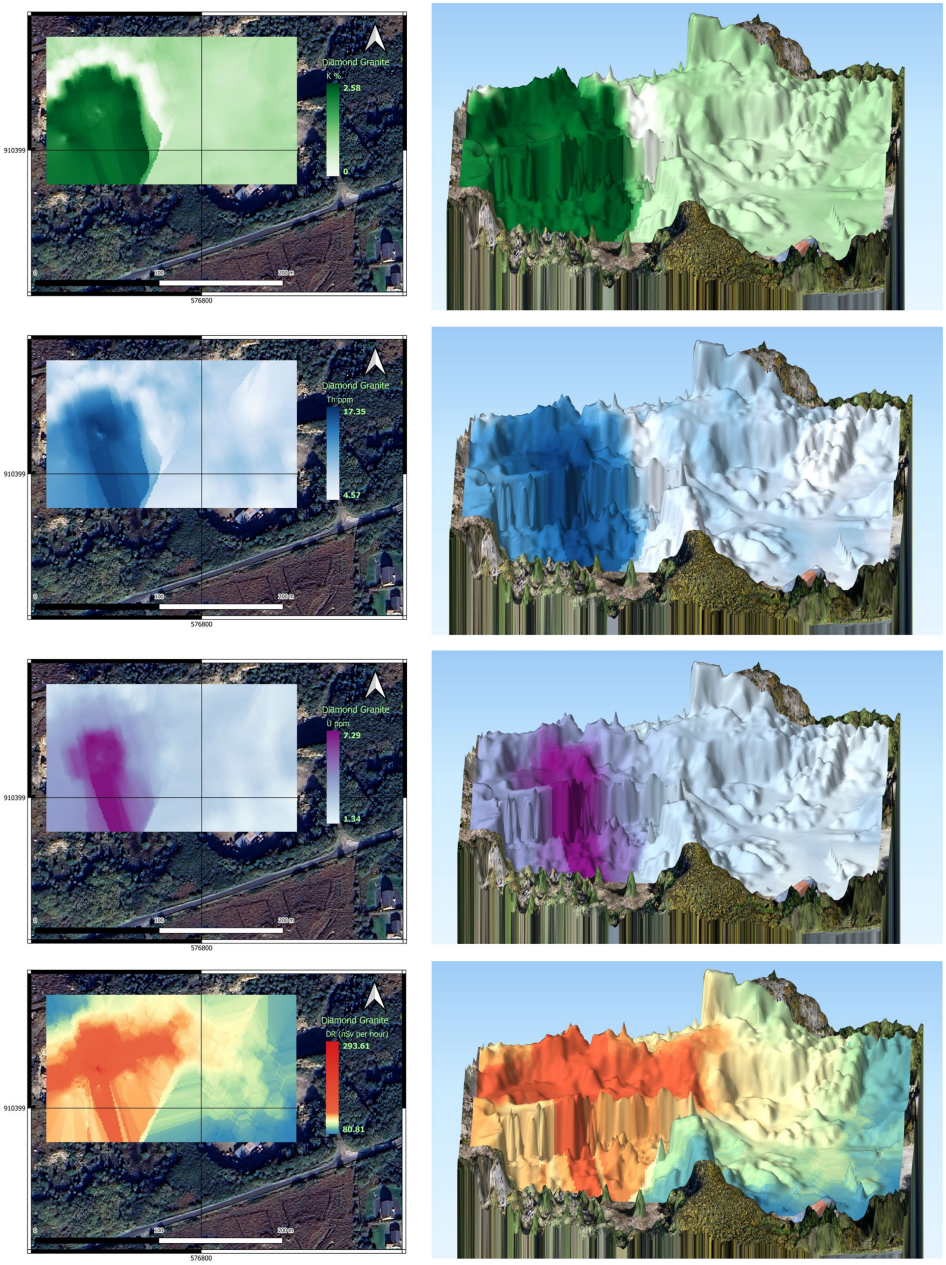
2D and 3D distribution maps of radionuclides at the Diamond Granite Quarry based on drone surveys.

Figure 8 shows an example of the produced 2D and 3D radionuclide distribution maps [i.e., U, Th, K and DR (dose rate)], for site D, the Diamond Granite Quarry. As mentioned earlier for site D, some areas in the south and eastern parts have undergone a high degree of alteration and therefore elevated values of potassium and uranium were observed. The range of radionuclide activities is consistent with the values obtained using the GT40 spectrometer operated in assay mode (see Table 3). It seems that values recorded on-site in the assay mode better indicate the radiation levels of the rocks than the results of tests conducted in the laboratory on the collected samples. To aim categorization of gamma exposure levels of the products from studied quarries, an approximate categorization of the dose rate from building material in low to high levels could be as follows (40);

- Low: 10–50 nSv/hr—Examples: wood, brick, concrete,
- Medium: 50–200 nSv/hr—Examples: granite, marble, sandstone,
- High: 200–1,000 nSv/hr—Examples: some types of ceramic tiles, some types of natural stone.

The dose from gamma radiation of the tested building material in the studied quarries is mainly at a low level except for altered zones where the dose might be medium to high. The majority of products in these quarries are extracted from fresh outcrops therefore as mentioned in section (3.2.1) Excessive gamma exposure from the products of these quarries was not expected. Modelling of the dose received from possible inhalation of radon and thoron of these materials is an ongoing task which is part of the second phase of the project to assess natural radioactivity and radon/thoron exhalation rates of Irish building and construction material.

## 4 Discussion

The Tellus radiometric data provides information on the concentration of radioactive elements in soil and rocks. Numerous studies have validated the use of these data for natural radioactivity and radon/thoron mapping applications (41, 42). This information can be leveraged for environmental monitoring and risk assessment, identifying areas with high levels of natural radioactivity and radon/thoron gas emissions. However, the data has limitations, such as relatively low spatial resolution, which must be considered when using it for decision-making purposes.

Drone-borne radiometric surveys can potentially overcome some of these limitations by providing higher resolution and accuracy. Drones equipped with radiation detectors can fly over areas of interest and collect data on the concentration of radioactive elements in soil and rocks at a much finer scale than ground-based surveys or satellite imagery. This can provide more detailed information for environmental monitoring, risk assessment, and mineral exploration. Additionally, drone surveys can cover areas that are difficult to access or too dangerous for human surveyors.

A portable gamma detector like a GT-40 spectrometer can be used to collect data at the same locations as the drone-borne radiometric survey, enabling us to compare the results and ensure they are consistent. This helps to verify the accuracy of the drone survey data and can serve as a backup tool to validate the drone survey results and improve the overall accuracy of radiometric data collection. While this approach can provide high-resolution data on the distribution of natural radioactivity in an area, it is still necessary to conduct actual indoor thoron measurements to produce accurate geogenic and indoor radon/thoron prediction models. This is because the concentration of thoron in indoor environments is influenced by various factors such as ventilation rate, building materials, and occupancy patterns, which cannot be accurately captured by remote sensing techniques alone.

Actual indoor thoron measurements are also necessary to recalculate the real dose from thoron and radon. The dose of thoron depends on its concentration in indoor air, which can vary significantly from one building to another. By measuring the actual thoron concentration along with radon activity in indoor environments, more accurate dose estimates can be obtained, which is important for assessing health risks and developing appropriate mitigation strategies. We found that radon anomalies are more pronounced in certain sedimentary and metamorphic rocks, such as shales and quartzites, due to their higher uranium content. Although volcanic rocks can also exhibit elevated radon potential, they typically have lower uranium concentrations.

### 4.1 Comparison of the potential maps with the EPA geogenic radon map

The EPA recently published a radon map of Ireland (43) which incorporates multiple geogenic factors and indoor radon measurements to produce the radon potential map. This work followed a similar methodology published in earlier research by Elío et al. (44, 45). A simple comparison of the map produced in the current paper with the EPA’s radon map shows a good spatial correlation when the areas of high radon and thoron are combined. In other words, for most parts of Ireland, the map we produced is similar to the EPA’s map, however, the new map presented here can distinguish between radon and thoron potentials. It is important to add that our map was not able to predict the geogenic potential radon and thoron potentials for the areas where Tellus data has not yet been provided.

Although different methodologies were adopted to develop both maps, it is reasonable to expect that similarity as methods used to create both maps may have accounted for similar geological and environmental factors that influence the distribution of natural radioactivity. However, it is important to note that combining radon and thoron potentials into a single map may not provide a complete picture of the health risks associated with natural radioactivity. Radon and thoron have different properties from dose and exposure points of view and effects on human health, and exposure to each element should be assessed separately. Therefore, it may be necessary to use both maps in conjunction with indoor measurements (both radon and thoron) and other data sources to fully understand the potential health risks in a given area.

## 5 Conclusion

We anticipate that thoron may present greater radiation exposure and potential health risks compared to radon in certain areas of Ireland. Therefore, monitoring indoor thoron levels alongside radon is essential for accurately assessing indoor air quality and identifying potential health hazards. The integration of drone-borne 2D and 3D surveys, Tellus radiometric data, and portable spectrometers can yield high-resolution information on the distribution of natural radioactivity in homes, planning sites, quarries, and workplaces. This data enables the identification of areas with high geogenic radon and thoron potential, which is critical for public health, environmental monitoring, and radiation risk assessments. By providing updated information on the doses received by occupants, it is possible to take proactive measures to mitigate health risks associated with radon and thoron, enhance our understanding of natural radioactivity’s environmental impact, and facilitate mineral exploration at a higher resolution.

In this paper, we utilized Tellus data to develop preliminary models for predicting geogenic radon and thoron potentials separately and to estimate the degree of thoron interference. However, to create robust models, it is imperative to conduct actual indoor thoron measurements and incorporate these as additional response variables. This will provide a more comprehensive understanding of the distribution of indoor radon and thoron potentials. For large-scale surveys, such as national radon monitoring, we recommend using up-to-date radon-thoron discriminative detectors, like Electret Ion detectors. Measurements that do not differentiate between radon isotopes can lead to inaccurate risk estimates, so special attention must be given to thoron concentration alongside radon measurements. Additionally, we have identified several quarries that may produce building and construction materials with elevated radon and thoron potentials. This information is crucial for developing strategies to reduce exposure levels, particularly to thoron, and for enhancing safety practices in construction.

## Data Availability

The raw data supporting the conclusions of this article will be made available by the authors, without undue reservation.

## Footnotes

^1^ www.gsi.ie
